# Y chromosome microdeletions frequency in idiopathic azoospermia, oligoasthenozoospermia, and oligospermia 

**Published:** 2017-11

**Authors:** Delnya Gholami, Hamideh Jafari-Ghahfarokhi, Maryam Nemati-Dehkordi, Hossien Teimori

**Affiliations:** 1 *Cellular and Molecular Research Center, Basic Health Sciences Institute, Shahrekord University of Medical Sciences, Shahrekord, Iran.*; 2 *Department of Gynecology, Medical Faculty, Shahrekord University of Medical Sciences, Shahrekord, Iran.*

**Keywords:** Male infertility, Y-chromosome microdeletions, Azoospermia factors

## Abstract

**Background:**

Genetic factors are candidates for about 30% of male infertility with sperm production-related abnormalities. Y chromosome microdeletions are responsible for around 10% of male infertility. These microdeletions generally occur in azoospermia factor on the Yq. That is often associated with the quantitative reduction of sperm.

**Objective:**

The aim of this cross-sectional study was to determine the frequency of Yq microdeletions among idiopathic azoospermic, oligoasthenozoospermic, and oligospermic men in Shohada infertility center, Chaharmahal va Bakhtiari province.

**Materials and Methods:**

A total of 81 idiopathic azoospermic, oligoasthenozoospermic, and oligospermic infertile men were selected as cases and 81 fertile men assigned to control group. For molecular investigations, 13 sequence-tagged site markers were chosen from azoospermia factor (AZF) region for detection of Y chromosome microdeletions and amplified by two separate multiplex-polymerase chain reaction. The relationship between the AZF microdeletions and incidence of male infertility in the family, consanguineous parents, smoking, and the levels of reproductive hormones among infertile men were investigated.

**Results:**

The total frequency of the microdeletions was 6.17% (2 cases in azoospermic, 3 cases in oligoasthenozoospermic subgroups, and none in the oligospermic participants and the control group). Most deletions (3.7%) were seen in the AZFb followed by the AZFc (2.46%) and none in AZFa. No significant association was seen between the microdeletions and clinical characteristics.

**Conclusion:**

Although the frequency of Yq chromosome microdeletions in Chaharmahal va Bakhtiari province is lower than the mean frequency of Iran, the frequency is comparable to those reported by some studies in Iran.

## Introduction

Infertility refers to decline in or lack of ability to conceive after 12 months or more of regular unprotected sexual intercourse (1). Currently, infertility is considered a main public health issue with significant psycho-socioeconomic impacts (2). Worldwide, 10-15% of couples are estimated to be affected by infertility, and men are found to be responsible for infertility in 40% of such couples (3). Male infertility is a multifactorial condition involving a wide spectrum of disorders (4). It has been estimated that more than 30% of male infertility is due to sperm production-related abnormalities caused by a genetic defect (5). Around 40% of factors for male infertility is unknown that is referred to as idiopathic factors (6).

Human Y chromosome plays a pivotal role in male fertility (7). Several studies have demonstrated that one or more genes located on the long arm of the Y chromosome are most probably involved in the complex process of spermatogenesis. AZF region can be divided into four subsidiary non-overlapping subregions namely AZFa, AZFb, AZFc, and AZFd (8). The AZFa locus is located in the Y chromosome proximal (Yq11) region (9). AZFa deletion occurs due to recombination of two directions, 10-kb repetitions with 800-kb distance from each other complete AZFa deletion causes Sertoli cell-only syndrome (9, 10).

AZFb is located in the male-specific region of the Y chromosome (MSY) (9). *RBMY*, *PRY*, and *CDY2* are some of the protein-encoding gene families in the AZFb region that expressed only in the testes (11). The AZFc locus has been mapped into distal Yq11 (7). Deletions in the AZFc include widely various phenotypes (12). AFZd, located between AZFb and AZFc have been proposed (13). Patients with AZFd microdeletions may have mild oligospermia or normal sperm count but abnormal sperm morphology (14). Deletions in these loci are associated with male infertility (15). The Y chromosome microdeletions are the second leading cause of spermatogenic failure after Klinefelter syndrome (16). 

Screening to diagnose Y chromosome microdeletions in infertile men is conducted mainly by using polymerase chain reaction (PCR) in the leukocytes based on sequence-tagged site (STS) (17, 18). The STS is a known short DNA sequence whose location in the genome is mapped (19). The accessibility of the nucleotide sequence of the MSY makes it possible to select the best PCR markers from an STS pool (20). According to the guideline of the European Academy of Andrology and the European Molecular Genetics Quality Network, six STSs (SY84 and SY86 in the AZFa region, SY127 and SY134 in the AZFb region , and SY254 and SY255 in the AZFc region) are used to detect complete deletion of AZF that consist of two STS sets in each AZF and are able to diagnose over 95% of the deletions (18, 21). 

The present study was conducted to investigate the frequency of microdeletions of these regions using multiplex PCR in men with idiopathic azoospermia, oligospermia, and oligoasthenozoospermia and then the association of these microdeletions with the incidence of male infertility in the family, consanguinity parents, smoking (cigarette and its derivatives), and changes in the levels of the reproductive hormone in Chaharmahal va Bakhtiari province.

## Materials and methods

In this cross-sectional study, 81 men with idiopathic azoospermic, oligospermic, and oligoasthenozoospermic infertile men as the case group and 81 healthy fertile men, as controls referred to the Shohada Fertility center of Shahrekord from May 2015 to May 2016. The Power and Sample size software was used to calculate the sample size. were investigated. Data on family history including male infertility in the first- and second-degree relatives, consanguinity parents, and the use of alcohol, tobacco, and other drugs were collected by interview and medical data including the levels of testosterone, prolactin, luteinizing hormone (LH), and follicle-stimulating hormone (FSH), and the results of chromosomal analysis were drawn from their medical files.

History of mumps in childhood, radiotherapy, chemotherapy, testicular trauma, varicocele, testicular tissue disorders, cryptorchidism, mutations in the CFTR, defects in the androgen receptor, and karyotype abnormalities such as Klinefelter syndrome were considered exclusion criteria. The participants were blind to the cause of their infertility, either acquired or genetic. The control group consisted of fertile men with children, who had no changes in seminal plasma parameters, no abnormalities in the reproductive system and no sex hormones abnormalities. 5 ml peripheral venous blood samples were taken from all participants and collected into Ethylenediaminetetraacetic acid (EDTA)-containing tubes. The samples were stored at -20^o^C temperature till conducting molecular tests.

**DNA extraction**

To extract DNA, first, cell buffy coat was isolated by rinsing with red cell lysis buffer three times and centrifugation at 6000 rpm for 5 min at 4^o^C. Then, pure DNA was isolated from the resulting buffy coat using a commercially available kit (CinnaGen Co., Iran) according to the manufacturer's instructions and investigated quantitatively and qualitatively using NanoDrop.

**Designing primers and selecting STS**

Thirteen STSs were selected according to the European Academy of Andrology and European Molecular Genetics Quality Network guidelines: SY82, SY84, and SY86 in the AZFa, SY272, SY134, SY142, and SY127 in the AFZb, and SY254, SY255, and SY277 in the AZFc, SY133 in the AZFd, and SY11 and SY238 in Yp. SY14 that is located on SRY was used as internal control. A number of appropriate primers were selected for these STSs using the National Center for Biotechnology Information database and checked and blasted for specificity and efficiency using the MFE primer database (Table I).

**Multiplex PCR**

All above mentioned STSs were amplified by two separate reactions as multiplex by a thermocycler (Thermal GeneAtals Cycler, Astec). For each multiplex-PCR, 1 µl (50-100 ng) of DNA was extracted from peripheral blood cells was added in 15 µl reaction mix that included 1.25 µl amplification buffer 1x (KBC kit), 0.5-0.75 µl MgCl_2_ (KBC kit), 0.5 µl dNTP (KBC kit), 0.1 µl Taq DNA polymerase (1 unit, KBC kit) and 0.2-0.7 µl (10-20 pmol) of each primer.

**PCR program**

Multiplex-PCR I: Amplification of DNA by 5 cycles with 95^o^C for 60 sec, 56^o^C for 60 sec, and 72^o^C for 60 sec and by 26 cycles with 95^o^C for 40 sec, 56^o^C for 35 sec, and 72^o^C for 40 sec. For multiplex-PCR II: Amplification of DNA by 7 cycles with 95^o^C for 60 sec, 62^o^C for 60 sec (touch-down with -0.5^o^C temperature difference), and 72^o^C for 60 sec and by 26 cycles with 95^o^C for 40 sec, 59.5^o^C for 40 sec, and 72^o^C for 40 sec including an initial denaturation step at 95^o^C for 5 min, and a final extension step at 72^o^C for 5 min for both of multiplex-PCR. sY14 was used as an internal control. 

**Figure 1:**
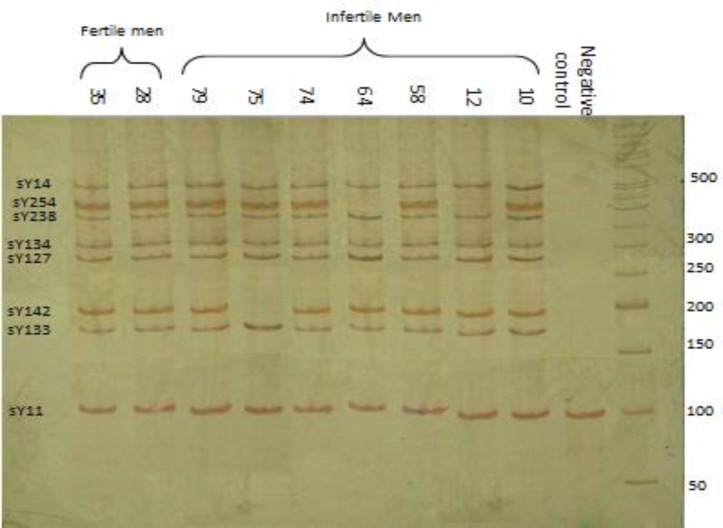
The results of multiplex-PCR; men no. 12 and 64 with deletions in the SY254 and no. 75 with a deletion in SY142; negative control is female DNA with SY11 amplification; this homologous STS is located on X chromosome. The last two bands represent healthy men

Normal fertile men were considered as normal control and also healthy women as the negative control. sY11 located at Yp and this homologous STS is located on X chromosome that amplified in healthy women (Figure 1). All products were run on a polyacrylamide gel. To better separate, the bands from each other, gel 10% and 45 mA current were used.

**Ethical consideration**

Study protocol was approved by the Ethics Committee of the Shahrekord University of Medical Sciences (no. IR.SKUMS.REC.1394.94) and all ethical considerations including voluntary participation in the study and participats’ confidentiality were observed. 

**Statistical analysis**

Statistical analysis was carried out by SPSS software (Statistical Package for the Social Sciences, version 23.0, SPSS Inc, Chicago, Illinois, USA) using chi-square test. Differences were considered statistically significant if p<0.05.

## Results

**Figure 2:**
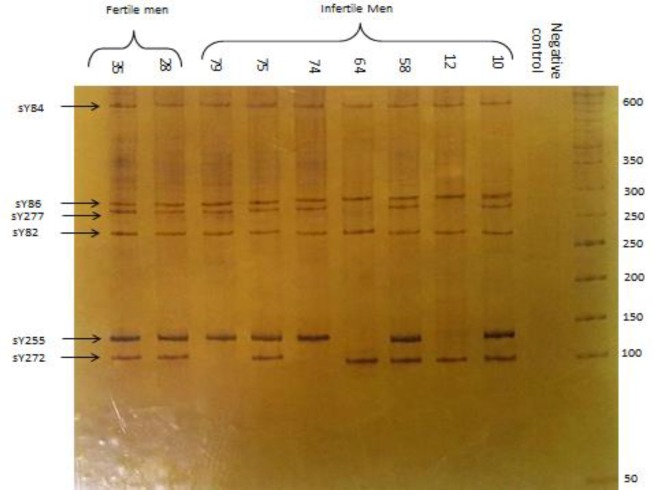
The results of multiplex-PCR; men no. 12 and 64 with deletions in the sY255 and sY277 no. 74 and 79 with a deletion in SY272; negative control is female DNA without any band amplification; the last two bands represent healthy fertile men

The age range of the infertile participants was 21-47 yr old. The control group was age-matched with the infertile participants. Of the infertile participants, 30 were azoospermic, 15 oligospermic, and 36 were oligoasthenozoospermic (Table II). All 13 STSs were conducted by multiplex PCR for both infertile and healthy participants. A total of nine deletions in five out of 81 participants were observed. Two participants (men no. 12 and 64) had three deletions in the SY254, SY255, and SY277, which are located on *Deleted in Azoospermia (DAZ)* gene in the AFZc; two participants (men no. 74 and 79) had one deletion in the SY272; and one participant (men no. 75) had one deletion in the SY142. The negative control was female DNA with SY11 amplification; this homologous STS is located on X chromosome (Figure 1, 2).

**Association between microdeletion and infertility phenotypes**

**Figure 3:**
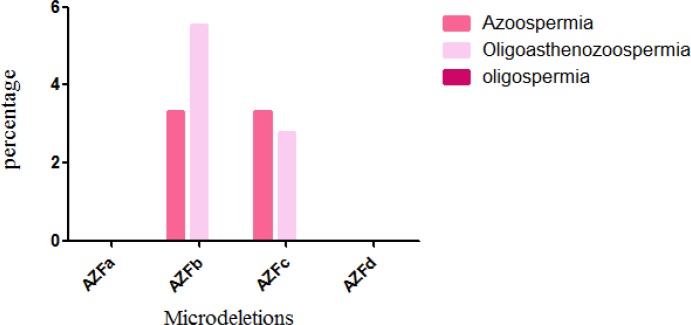
Percentage of patients with Y chromosome microdeletions.

The frequency of deletions was 0% in the AFZa, 3.7% (3/81) in the AFZb, and 2.46% (2/81) in the AFZc. Overall, 6.17% of all the participants had at least one deletion in the Yq, and no deletion was seen in the oligospermic participants and the control group. The frequency of microdeletions in the azoospermic, oligoasthenozoospermic, and oligospermic participants was 6.67%, 8.33% and 0%, respectively (Table III, Figure 3).

**Association between microdeletions and clinical and family parameters**

Fifteen participants were found to have hormonal changes. Most changes were seen in LH and FSH. The frequency of hormonal changes in the azoospermic, oligoasthenozoospermic, and oligospermic participants was 30%, 11.11%, and 13% respectively. Out of five men with microdeletions in the Y chromosome, only one was found to have hormonal changes (changes in LH and FSH levels), two reported to have male infertility in their family, one had consanguinity parents, and one was a smoker (Table III). The χ^2^ test indicated no significant association between these deletions and clinical and family variables (p=0.33). while, the null hypothesis stating that there is a significant association between the microdeletions and the above-mentioned variables, was rejected.

**Table I:** Studied sequence-tagged sites and primer sequences

**Groups**	**STS**	**Locus**	**Primer sequences**	**MW (bp)**
I	sY14	SRY	F: GAATATTCCCGCTCTCCGGA R: GCTGGTGCTCCATTCTTGAG	470bp
I	sY134	AZFb	F: GTCTGCCTCACCATAAAACG R: ACCACTGCCAAAACTTTCAA	303bp
I	sY142	AZFb	F: AGCTTCTATTCGAGGGCTTC R: CTCTCTGCAATCCCTGACAT	196bp
I	sY238	AZF	F: AACAAGTGAGTTCCACAGGG R: GCAAAGCAGCATTCAAAACA	358bp
I	sY11	AZF	F: CATGTGAACAGTACACATCTCTG R: ATAATAATTTTCTACACGCAGTTCC	103bp
I	sY133	AZFd	F: ATTTCTCTGCCCTTCACCAG R: TGATGATTGCCTAAAGGGAA	177bp
I	sY127	AZFb	F: GGCTCACAAACGAAAAGAAA R: CTGCAGGCAGTAATAAGGGA	274bp
I	sY254	AZFc	F: GGGTGTTACCAGAAGGCAAA R: GAACCGTATCTACCAAAGCAGC	380bp
II	sY82	AZFa	F: ATCCTGCCCTTCTGAATCTC R: CAGTGTCCACTGATGGATGA	264bp
II	sY84	AZFa	F: GCTGAGGAGTTGTGGAGACC R: GCAAGGACATTCCAGGGTTA	642bp
II	sY86	AZFa	F: GTGACACACAGACTATGCTTC R: ACACACAGAGGGACAACCCT	318bp
II	sY255	AZFc	F: GTTACAGGATTCGGCGTGAT R: CTCGTCATGTGCAGCCAC	124bp
II	sY277	AZFc	F: GGGTTTTGCCTGCATACGTAATTA R: CCTAAAAGCAATTCTAAACCTCCAG	312bp
II	sY272	AZFb	F: GGTGAGTCAAATTAGTCAATGTCC R: CCTTACCACAGGACAGAGGG	98bp

STS: Sequence-tagged site

SRY: Sex-determining region Y

AZF: Azoospermia factor

MW: Molecular weight

**Table II:** The groups of the study and the number of men in each group

**Groups**		**Number**	**Family history of infertility**	**Consanguinity parents**	**Changes in sex hormones**	**Smoker**
Case						
	Azoospermia	30	5	9	9	18
	Oligozoospermia	15	4	8	2	12
	Oligoasthenozoospermia	36	12	13	4	16
Control						
	Normospermia	81	0	40	0	40

**Table III:** Some features of 5 patients with Y chromosome microdeletions

**Patient case No**	**Clinical phenotypes**	**STS deletion**	**Locus**	**Age**	**Changes in sex hormones**	**Family history of infertility**	**Consanguinity parents**	**Smoker**
12	Oligoasthenozoospermia	sY277,sY255,sY254	AZFc	34	Normal	Father	-	-
64	Azoospermia	sY277,sY255,sY254	AZFc	47	High FSH	-	-	-
74	Oligoasthenozoospermia	sY272	AZFb	34	Normal	-	-	-
75	Azoospermia	sY142	AZFb	37	Normal	-	-	-
79	Oligoasthenozoospermia	sY272	AZFb	28	Normal	Uncle	+	+

AZF: Azoospermia factor

STS: Sequence-tagged site

**Table IV:** Studies in Iran with different frequencies

**Studies**	**Province**	**Number of cases**	**Frequency YCM (%)**
Saliminejad, *et al.* (2012) (32)	Tehran	115	1.74
Etemadi, *et al.* (2013) (33)	Hamedan	56	1.87
Shaveisi zadeh, *et al.* (2017) (34)	Kermanshah	108	4.6
Akbari asbagh, *et al.* (2003) (35)	Tehran	40	5
Asadi, *et al.* (2016) (36)	Tehran	1885	5.2
Motovali-bashi, *et al* (2015) (37)	Esfahan	100	7
Masoudi, *et al* (2016) (38)	Fars	81	7.4
Zaimy, *et al* (2013) (2)	Yazd	50	8
Torfeh, *et al* (2015) (39)	Esfahan and East Azerbaijan	100	8
Keshvari, *et al* (2011) (40)	Khorasan Razavi	47	8.51
Kalantar, *et al* (2011) (41)	Yazd	90	8. 8
Mirfakhraie, *et al* (2010) (42)	Tehran	100	12
Khatami, *et al* (2013) (43)	Khozestan	84	12
Sheikhha, *et al* (2013) (44)	Yazd	25	20
Omrani, *et al.* (2006) (45)	West Azarbaijan	99	24
Akbarzadeh, *et al* (2013) (46)	East Azarbaijan	94	51.06
Malekasgar, *et al* (2008) (47)	Gilan	50	52

YCM: Y-Chromosome microdeletion

## Discussion

In the present study, the total frequency of the microdeletions was 6.17% (approximately 6.67% of the azoospermic participants and 8.33% in oligoasthenozoospermic ones). Most (3.7%) deletions were seen in the AZFb followed by the AZFc (2.46%). No significant association was seen between the microdeletions and clinical and family parameters.

The Y chromosome microdeletions are the second leading cause of spermatogenic failure after Klinefelter syndrome (16). The frequency of the Y chromosome microdeletions is between 1% and 55% across the world (7, 8), but the most reported frequency of these microdeletions is less than 15% (8). Deletion in AFZc (around 80%) is the most prevalent and frequent deletion followed by those in AZFb (1-5%), AZFa (0.5-4%), and AZFbc (1-3%) (22). The current study's findings are consistent with some studies. However, most studies have reported that deletions occur most frequently in the AFZc and in azoospermic men (5, 23-29). Inconsistent with these findings, the current study showed that the deletions occurred most frequently in the AZFb and in the oligoasthenozoospermic men.

People with deletions in the AZFc and partial deletions in AZFb manifest a wide spectrum of infertility phenotypes from normal fertility to oligospermia and azoospermia (8, 12). All deletions which were observed in this study occurred in the AZFc or partially in the AZFb. According to this evidence, probably the higher frequency of deletions in oligoasthenospermia compared to azoospermia can be explained. It is noteworthy that we can attribute this difference in the frequency of deletions to difference in ethnicities.

Overall, the frequency of microdeletions is widely various (1.74-52%) in different populations of Iran (Table IV). A meta-analysis conducted on infertile patients in Iran reported the frequency of these microdeletions 12.1% (30) that is partly consistent with frequency reported for East Asia populations (10%) (16). However, the total frequency of a small proportion of these microdeletions in European population is higher than 8% (31).

Some studies indicate that in different countries and regions, both the frequency of Y chromosome deletions and the regions of deletions are different (2, 16, 32-47). Inconsistency in the findings can be explained by different sample size, ethnicities, inclusion criteria, methods, and even the types and numbers of the used STSs. Indeed, ethnicity can be a determinant of the types and frequency of the Y chromosome microdeletions in infertile men in different populations (48). Comprehensive these studies on men from different ethnicities and various geographical populations provide valuable information about the significance of these deletions (11, 16, 30, 31).

The levels of sex hormones such as LH, FSH, prolactin, testosterone in the infertile men were markedly higher than those in the healthy men, and the frequency of hormonal changes in the azoospermic men was higher than that in other subgroups of the infertile men. However, a significant difference in the levels of FSH, LH, testosterone, and prolactin was not seen among patients with microdeletions in the AZF and those without these microdeletions. This finding is consistent with previous studies. However, Zhang *et al* Reported that prolactin levels decreased in infertile men with a microdeletion in the Yq (4). These findings imply that microdeletions in AZF in infertile men cannot be predicted by the levels of FSH, LH, prolactin, and testosterone. To predict such microdeletions, further studies with larger sample size should be conducted. 

In the current study, there was no significant association between Y chromosome microdeletions and the existence of other infertile men in the family. The Y chromosome microdeletions are either inherited through paternal germline or occur as de-novo events. Many of the Y chromosome microdeletions are originated from de-novo events (49). Therefore, this finding cannot be considered an unexpected one. Most likely, this event occurs before fertilization, although deletion can also occur after fertilization (7).

The mechanism of the Y chromosome microdeletions has not yet been clearly explained. Because unlike autosomal chromosomes, a limited number of the Y chromosome's parts are paired with the X chromosome and no recombination occurs in the AZF regions, it has been suggested that the Y chromosome microdeletion is most likely due to the presence of highly repetitive DNA elements that launch abnormal intrachromosomal recombination. Certain genetic and environmental factors may predispose some men to produce a higher proportion of sperm with de-novo deletions (9). Therefore, it is essential to study genetic and environmental background to investigate the origins of the microdeletions. To do this in the present study, we investigated the participants' family history and environmental conditions such as the presence of other infertile men in the family, the consanguinity parents, and smoking and using tobacco derivatives to study their potential association with the microdeletions. However, because the genetic information about other family members was not available and the number of the samples was small, no significant association was seen. Therefore, studies with larger sample size and genetic investigations should be conducted to examine such variables. Screening for Y chromosome microdeletions has diagnostic, prognostic, and preventive value and therefore has been much frequently investigated in different regions of the world (7). Treating infertility by means of assisted reproductive technology (ART) such as in vitro fertilization and ICSI can lead to the transfer of the Y chromosome microdeletions to male children and thus perpetuate the problem of infertility in the next generation (2, 50, 51). 

Association between the Y chromosome microdeletions and 46, XY/45, X0 mosaicism or isodicentric Y chromosome has already been demonstrated. In addition, it has been suggested that the Y chromosome microdeletions are likely to lead to loss of mitotic Y chromosome and gonadal disorders in children through inducing Y chromosome instability. Screening of microdeletions and preimplantation genetic diagnosis, as a treatment of choice for these disorders, should be studied because of the risk of transmission of such conditions to children (8).

There has been much debate about the outcomes of treating infertile men with the Y chromosome microdeletions. In some studies, similar findings in patients with or without the Yq microdeletion have been observed, and in some others, certain differences particularly in fertilization between these two groups of infertile patients, have been noted. However, the side effects of the Y chromosome microdeletions on the ICSI outcomes have not yet been extensively studied (52). In addition, a study demonstrated that ICSI in oligospermic men with a microdeletion in the Y chromosome AZFc subregion resulted in lower quality fertilization and poorer embryo which the couples should be informed about (35). Therefore, it is necessary to study the Yq microdeletions to gain knowledge about such difficulties and estimate ART success rate. 

The existence of sperm is a prerequisite for couples undergoing ICSI. Some studies have shown that mature sperm cells can be detected in men with a microdeletion in the AZFb and the AZFc and therefore such men can achieve fertility (7, 12). Moreover, the AZFc microdeletion can be associated with the declined production of sperm over time (12, 53). 

Therefore, freezing seminal plasma in early adulthood can be recommended for men with the AFZc microdeletion (12). Studying the microdeletions can be helpful in this regard. Generally, identifying and diagnosing microdeletions can help physicians to prevent patients from undergoing experimental and often costly treatments for infertility and also provides information that makes it possible to find sperm in azoospermic men's testes and freeze oligospermic men's sperm (7). More clearly, it is highly important to investigate these microdeletions to detect microdeletions in question before undergoing ICSI, contribute to treatments for infertility, conduct genetic experiments on azoospermic and oligospermic men, screen sperm before donating it to the sperm bank and population genetics, and enhance the speed of discovering and identifying the Y chromosome microdeletions in different countries and regions (54).

In this study, 14 PCR reactions were summarized in two multiplex PCR and therefore time and costs were significantly saved. In addition, all four AZF regions were covered by selecting appropriate STSs. Besides that, the maximum accuracy of the multiplex PCR was ensured using SY14 as the internal control and SY11 that is located in the Yp and amplified in the female DNA samples (as the negative control). It is also worth mentioning that isolating buffy coat from the blood samples was extremely useful to obtain high quality and pure DNA samples. 

## Conclusion

Taken together, it can be argued that studied microdeletions in this study play a significant role in the etiology of idiopathic infertile men in Chaharmahal va Bakhtiari province. However, further studies with larger sample size and more diverse STSs should be conducted to definitely confirm this finding. In addition, multiplex PCR can be used to study the 13 STSs because of ease, short duration, and low cost.

Given the significance of studying microdeletions in azoospermic, oligospermic and oligoasthenozoospermic infertile men, the high frequency of these microdeletions in the male population of Iran, and the growing population of infertile men, all azoospermic, oligospermic, and oligoasthenozoospermic infertile men and infertile couples who are seeking to undergo in vitro fertilization and ICSI are recommended to gain knowledge about these microdeletions.
